# CD103+ intraepithelial T cells in high-grade serous ovarian cancer are phenotypically diverse TCRαβ+ CD8αβ+ T cells that can be targeted for cancer immunotherapy

**DOI:** 10.18632/oncotarget.12077

**Published:** 2016-09-16

**Authors:** Fenne L. Komdeur, Maartje C.A. Wouters, Hagma H. Workel, Aline M. Tijans, Anouk L.J. Terwindt, Kim L. Brunekreeft, Annechien Plat, Harry G. Klip, Florine A. Eggink, Ninke Leffers, Wijnand Helfrich, Douwe F. Samplonius, Edwin Bremer, G. Bea A. Wisman, Toos Daemen, Evelien W. Duiker, Harry Hollema, Hans W. Nijman, Marco de Bruyn

**Affiliations:** ^1^ University of Groningen, University Medical Center Groningen, Department of Obstetrics and Gynecology, The Netherlands; ^2^ University of Groningen, University Medical Center Groningen, Department of Medical Microbiology, The Netherlands; ^3^ University of Groningen, University Medical Center Groningen, Department of Surgery, The Netherlands; ^4^ University of Groningen, University Medical Center Groningen, Department of Pathology, The Netherlands

**Keywords:** tumor-infiltrating lymphocytes, high-grade serous ovarian cancer, CD103, TGF-β, cancer immunotherapy

## Abstract

CD103+ tumor-infiltrating lymphocytes (TIL) have been linked to specific epithelial infiltration and a prolonged survival in high-grade serous epithelial ovarian cancer (HGSC). However, whether these cells are induced as part of an ongoing anti-HGSC immune response or represent non-specifically expanded resident or mucosal lymphocytes remains largely unknown. In this study, we first confirmed that CD103+ TIL from HGSC were predominantly localized in the cancer epithelium and were strongly correlated with an improved prognosis. We further demonstrate that CD103+ TIL were almost exclusively CD3+ TCRαβ+ CD8αβ+ CD4- T cells, but heterogeneously expressed T cell memory and differentiation markers. Activation of peripheral T cells in the presence of HGSC was sufficient to trigger induction of CD103 in over 90% of all CD8+ cells in a T cell receptor (TCR)- and TGFβR1-dependent manner. Finally, CD103+ TIL isolated from primary HGSC showed signs of recent activation and dominantly co-expressed key immunotherapeutic targets PD-1 and CD27. Taken together, our data indicate CD103+ TIL in HGSC are formed as the result of an adaptive anti-tumor immune response that might be reactivated by (dual) checkpoint inhibition.

## INTRODUCTION

High-grade serous epithelial ovarian cancer (HGSC) is the most common cause of death from gynecological malignancies and is the fifth leading cause of female cancer death worldwide [1]. Despite successful initial treatment, 5-year survival is only 35% as almost all patients with advanced disease relapse. This poor prognosis has not improved in four decades and novel therapies for HGSC are urgently needed [2]. Herein, therapeutic exploitation of the immune system may be of value.

The immune system plays an important role in the development and control of HGSC, and the presence of tumor-infiltrating lymphocytes (TIL) in HGSC is associated with prolonged survival [3–5]. Indeed, infiltration of T cells, and particularly CD8+ cytotoxic T cells (CTL) is associated with an improved prognosis for HGSC patients [4–6]. Reactivating CTL is therefore an active area of investigation for therapy of HGSC. One issue that needs to be considered herein is the distribution of the CTL within the tumor. Whereas CTL localized in the tumor epithelium are associated with prolonged survival in HGSC patients, stromal CTL are not [6]. Therefore, developing immunotherapeutic strategies tailored to the specific biology of prognostically favorable intraepithelial CTL, whilst not activating potentially inflammatory stromal immune cells appears warranted.

The epithelial and stromal CTL populations in HGSC can be delineated by expression of the α_E_ integrin subunit CD103. In line with this, the total number of CD103+ TIL (irrespective of localization) was shown to be associated with improved prognosis [7]. By contrast, the total number of CD8+ TIL did not associate with an improved prognosis [7]. CD103+ TIL therefore represent an interesting population for therapeutic targeting. However, there are several remaining questions regarding CD103+ TIL that need to be addressed before these cells can be considered as key players in natural HGSC-directed immunological reactions.

CD103 is the prototypical marker for intraepithelial lymphocytes (IEL) and tissue-resident memory T cells (T_RM_), and has been linked to persistence of T cells involved in immune surveillance after pathogenic infection of (barrier) tissue. Indeed, CD103 was reported to be expressed on influenza-specific CD8+ T cells in the human lung, Epstein-Barr virus (EBV)-specific cells in human tonsil, and on vesicular stomatitis virus (VSV)-specific CD8+ T cells in a mouse model system of VSV infection of the brain [8–10]. Since ovarian/tubal tissue constitutes a de facto barrier, CD103+ T cells in HGSC might simply represent “native” (not tumor-specific) IEL or T_RM_ cells expanded due to the inflammatory reaction within the tumor. Such cells would have limited potential for therapeutic targeting in HGSC and would represent a poor biomarker for selection of patients for immunotherapy. By contrast, if CD103+ TIL in HGSC represent adaptive immune cells that are formed as part of an ongoing specific anti-cancer response in HGSC, these cells could be harnessed for adoptive cell therapy or targeted by e.g. immune checkpoint inhibitors.

To address these questions, we determined the ontogeny of CD103+ TIL in HGSC and explored whether these cells represent a promising target population for HGSC immunotherapy.

## RESULTS

### CD103 identifies intraepithelial CD8+ TIL with prognostic benefit in HGSC

**Figure 1:**
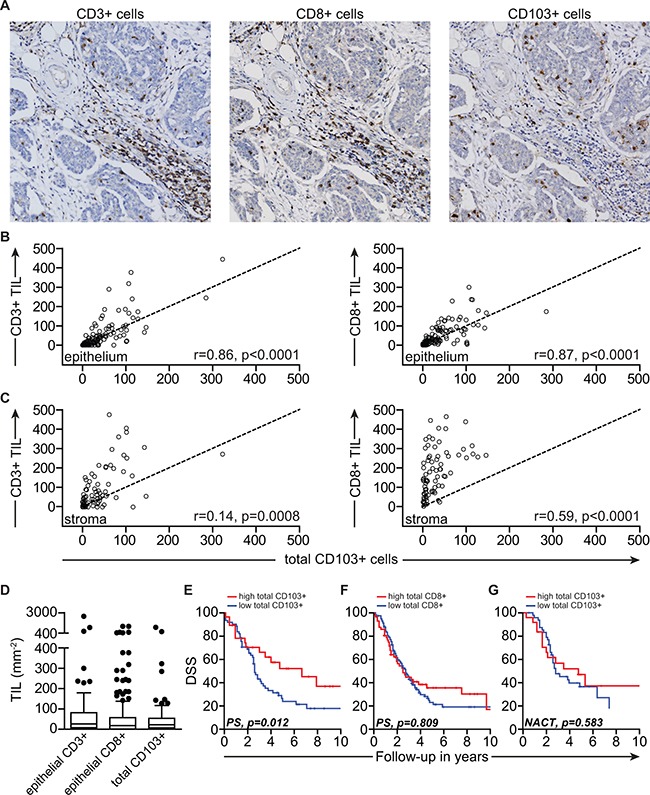
CD103 defines intraepithelial CD8+ TIL with prognostic benefit in HGSC patients **A.** Representative images of HGSC tissue cores with infiltration of CD3+, CD8+ or CD103+ cells. **B.** Correlation of total CD103+ cells per tumor core versus epithelial CD3+ cells or epithelial CD8+ cells. Line insert represents a hypothetical perfect correlation on the x-and y-axis. **C.** Correlation of total CD103+ cells versus stromal CD3+ cells or stromal CD8+ cells. Line insert represents a hypothetical perfect correlation on the x-and y-axis. Spearman's rank correlation coefficient was used to determine correlation between epithelial and stromal CD3+ and CD8+ TIL and total CD103+ TIL. **D.** Box-plots of epithelial CD3+, epithelial CD8+ and total CD103+ cell counts per 1 mm^2^ HGSC tissue. **E.** DSS (determined by Kaplan-Meier method with Log Rank test) of patients within the PS cohort according to infiltration of total CD103+ cells. **F.** DSS of patients within the PS cohort according to infiltration of total CD8+ cells. **G.** DSS of patients within the NACT cohort according to infiltration of total CD103+ cells.

First, we confirmed the recent reports on the prognostic benefit of CD103+ TIL in HGSC [7,11] in an independent cohort of advanced-stage HGSC patients treated with either primary surgery and adjuvant chemotherapy (PS; n=84), or neo-adjuvant chemotherapy followed by interval surgery (NACT; n=102) [12]. CD3+, CD8+ and CD103+ TIL were present in tumors from most patients and the distribution of CD3+ and CD8+ TIL in tumor epithelium and stroma was similar (Figure 1A). By contrast, CD103+ TIL were mainly found in the tumor epithelium (Figure 1A). Total CD103+ TIL numbers did not differ significantly between patients that received PS or NACT therapy (not shown), as reported previously for epithelial CD8+ TIL in this cohort [12]. In line with the observed epithelial distribution, total CD103+ TIL counts per mm^2^ in these patients correlated strongly to intraepithelial CD3+ and CD8+ TIL (r=0.86; p<0.0001 and r=0.87; p<0.0001, respectively), but weaker to stromal CD3+ and CD8+ TIL (r=0.14; p=0.0008 and r=0.59; p<0.0001, respectively) (Figure 1B and 1C). Moreover, the relative distribution of epithelial CD3+ or CD8+ and total CD103+ TIL counts were similar (Figure 1D). High total numbers of CD103+, but not total CD8+ TIL, were associated with a longer disease-specific survival (DSS) in patients treated with PS (p=0.012 and p<0.809, respectively) (Figure 1E and 1F). As previously reported for epithelial CD8+ TIL [12], total CD103+ TIL did not confer prognostic benefit in patients treated with NACT (p=0.583) (Figure 1G and Supplementary Figure S1). Taken together, our data confirm the restricted distribution of CD103+ TIL in the HGSC epithelium and suggest a CD3+ CD8+ phenotype for these cells.

### CD103+ TIL in HGSC are almost exclusively classical CD3+ CD56- TCRαβ+ CD8αβ+ CD4- T cells with heterogeneous differentiation status

The marker CD103 has been shown to define IEL in mucosal tissues, T_RM_ cells in non-lymphoid tissues, T_RM_ cells in lung cancer tissue, but also characterizes T cell clones that have been in recent contact with cognate epithelial tumor cell lines [13–19]. Therefore, to address whether CD103+ TIL in HGSC represent expanded IEL, T_RM_, or classical adaptive T cells that have migrated to HGSC tissue as a result of an ongoing immune response, we performed in-depth phenotyping on TIL isolated from primary HGSC tumors. As tissue dissociation and digestion may skew distribution of cell subsets and can even cause loss of cell surface antigens, we first studied CD103+ TIL infiltration in a side-by-side comparison of tissue and digests using 6 matched patient samples. Immunohistochemistry for detection of CD3, CD8 and CD103 was performed on full slides and complemented with flow cytometric analysis of the corresponding tumor digest. In all cases, CD103+ CD8+ cells were only observed in tumor digests when CD8+ and CD103+ cells could be detected in the tumor epithelium by immunohistochemistry (data not shown).

**Figure 2:**
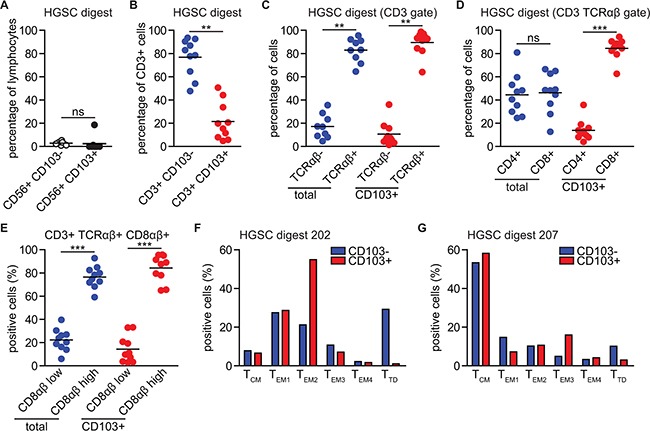
CD103+ TIL are almost exclusively classical CD3+ CD56- TCRαβ+ CD8αβ+ CD4- T cells with heterogeneous differentiation status HGSC tumor tissue was subjected to enzymatic digestion and analyzed by flow cytometry. **A.** Dot plot representing the percentage of CD56+ CD103- cells and CD56+ CD103+ cells within the total lymphocyte population isolated from HGSC tumor digests (N=10). **B.** Percentage of CD103- cells and CD103+ cells within the CD3+ cell population. **C.** Percentage of TCRαβ- and TCRαβ+ cells within the total CD3+ population or within the CD3+ CD103+ subpopulation. **D.** Percentage of CD4+ and CD8+ cells within the total CD3+ TCRαβ+ population or within the CD3+ TCRαβ+ CD103+ subpopulation. **E.** Percentage of CD8αβ_low_ and CD8αβ_high_ cells within the total CD3+ TCRαβ+ CD8αβ+ population or within the CD3+ TCRαβ+ CD8αβ+ CD103+ subpopulation. **F.** Bar graph of phenotypical subtype for CD103+ cells and CD103- cells derived from HGSC tumor digest #202. **G.** Bar graph of phenotypical subtype for CD103+ cells and CD103- cells derived from HGSC tumor digest #207. T_CM_: central memory T cell. T_EM_: effector memory T cell. T_TD_: terminally differentiated T cell. Differences were assessed by Mann-Whitney U or one-way ANOVA tests.

Marginal numbers of tumor-infiltrating NK-cells were observed by phenotyping the immune cell subpopulations in the tumor digests (n=10, Figure 2A), a finding in line with our previous reports. Furthermore, NK-cells expressed CD103 in only 1/10 analyzed digests at percentages >1% (Figure 2A). 10/10 patients had a detectable CD3+ CD103+ T cell infiltrate, although the majority of CD3+ TIL in the digests did not express CD103 (Figure 2B) (distribution of subsets per patient in Supplementary Figure S2). Importantly, CD103+ and CD103- TIL predominantly expressed the αβ chains of the T cell receptor (TCR) in conjunction with CD3 (Figure 2C). Moreover, no CD56 expression on these cells was detected (data not shown), excluding both yδ- and NKT-cells as dominant CD103-expressing T cells in HGSC.

Within the total CD3+ TCRαβ+ T cell population, both CD8+ and CD4+ cells were detected at roughly a 1:1 ratio (Figure 2D). By contrast, CD3+ TCRαβ+ CD103+ T cells were almost exclusively CD8+, with only marginal numbers of CD4+ cells detected (Figure 2D, p<0.0001). Notably, all cells expressing the CD8α chain also expressed the CD8β chain, and no cells were found to co-express CD4. As IEL are characterized by co-expression of CD4 and CD8α (but not CD8β), this suggests that CD103+ T cells in HGSC are not of an IEL origin. Interestingly, a subset of ~20-30% of all CD3+ TCRαβ+ CD8αβ+ cells expressed both CD8α and β chains at a lower level of roughly 50% of the mean fluorescent intensity, irrespective of CD103 expression (Figure 2E). The lower expression of CD8β has previously been reported in association with effector differentiation of T cells and suggests both CD103+ and CD103- T cells undergo similar differentiation in the HGSC microenvironment.

Finally, to address the resident memory status of the CD103+ TIL population, we analyzed expression of memory markers CD45RO, CCR7, CD27 and CD28 in conjunction with CD3, CD8 and CD103 expression in two additional HGSC digests (flow cytometric plots and gating strategy in Supplementary Figure S3). In line with our recent report on the differential expression of CD27 and CCR7/CD45RO in HGSC, TIL in these HGSC digests could be readily typed to various populations such as central memory (T_CM_), effector memory 1-4 (T_EM_1-4) and terminally differentiated (T_TD_) subtypes (Figure 2F and 2G). Nevertheless, T_CM_, T_EM_ and T_TD_ subtypes were observed in both CD103+ and CD103- CD8+ T cell populations and variation between CD103+ and CD103- cells was similar to variation between the two HGSC patient samples (Figure 2F and 2G). These data support a heterogeneous differentiation status of CD103+ TIL, not directly in line with a T_RM_ phenotype.

Our data therefore suggest CD103+ TIL in HGSC represent T cells of a classical CD3+ CD56- TCRαβ+ CD8αβ+ CD4- phenotype of varying differentiation. This phenotype is consistent with cells that have infiltrated as a result of an adaptive immune response.

### Activation in the presence of HGSC cells induces CD103 on peripheral blood CD8+ T cells through combined TCR- and TGFβR1-signaling

**Figure 3:**
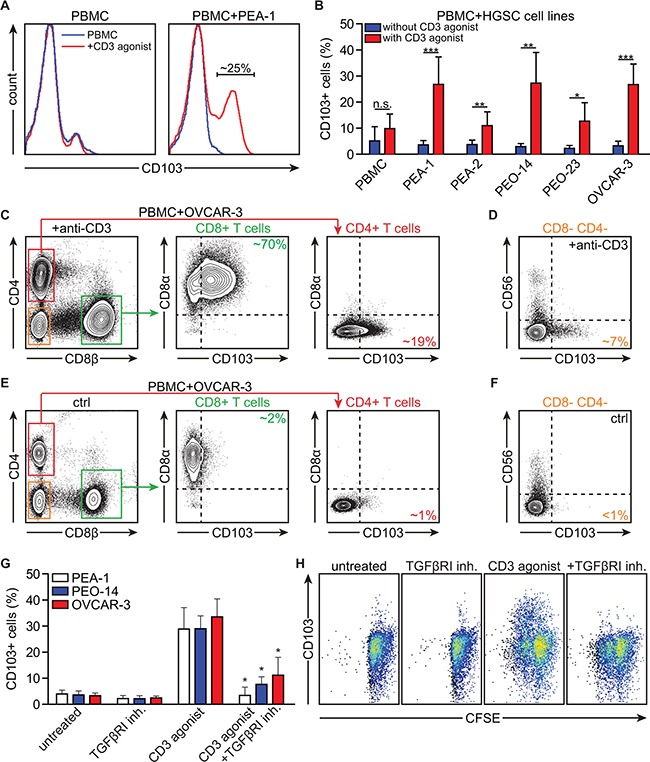
Activation in the presence of HGSC cell lines induces CD103 on peripheral blood CD8+ T cells through combined TCR and TGFβR1-signaling PBMCs were isolated and cultured in the presence of HGSC cell lines. **A.** Histograms representing CD103 expression on PBMCs after incubation of PBMCs with anti-CD3 agonistic antibody (CD3 agonist) in the presence or absence of HGSC cell line PEA-1. **B.** Bar graph representing the percentage of CD103+ cells after incubation of PBMCs with HGSC cell lines (PEA-1, PEA-2, PEO-14, PEO-23, OVCAR-3) in the presence or absence of CD3 agonist. **C.** Representative flow cytometry images of CD103 expression on the CD8+ T cell and CD4+ T cell populations after incubation with OVCAR-3 and CD3 agonist. **D.** Representative flow cytometry image of CD103 expression on the CD8-CD4- T cell population after incubation with OVCAR-3 and CD3 agonist. **E.** Representative flow cytometry images of CD103 expression on the CD8+ T cell and CD4+ T cell populations after incubation with OVCAR-3 without CD3 agonist (control). **F.** Representative flow cytometry image of CD103 expression on the CD8-CD4- T cell population after incubation with OVCAR-3 without CD3 agonist (control). **G.** Bar graph representing the percentage CD103 expressing cells after incubation of PBMCs with HGSC cell lines (PEA-1, PEO-14, OVCAR-3) and TGFβR1 inhibitor and/or CD3 agonist as indicated. **H.** Representative images of CFSE dilution showing T cell proliferation after incubation of PBMCs with OVCAR-3 and TGFβR1 inhibitor and/or CD3 agonist as indicated, showing CD103 expression on the y axis. Differences were assessed by Mann-Whitney U or one-way ANOVA tests.

The non-IEL, non-T_RM_ phenotype of CD103+ TIL in HGSC prompted us to speculate that induction of CD103 occurs as a natural consequence of peripheral blood T cell activation after infiltrating the HGSC epithelium. To assess this, we co-cultured peripheral blood mononuclear cells (PBMC) with HGSC cell lines and determined expression of CD103 in the presence or absence of a CD3 agonistic antibody (n≥3 donors). As exemplified in Figure 3A, activation of T cells in the presence of HGSC cell line PEA-1 induced expression of CD103 on a subset of ~25% of PBMC. Similar percentages were observed in the presence of a panel of HGSC cell lines, but not when T cells were activated alone (Figure 3A and 3B; n≥3 donors). In line with the *ex vivo* phenotyping data, co-culture of PBMC with OVCAR-3 cells predominantly induced CD103 on T cells of a CD8αβ+ phenotype, with much lower percentages observed in CD4+ cells (Figure 3C and 3E). A small subset of CD56- CD8- CD4- lymphocytes also upregulated CD103 in response to stimulation with anti-CD3 agonistic antibody in this setting (Figure 3D and 3F), although the exact identity of these cells remains unclear. Of note, T cell proliferation did not correlate directly with the induction of CD103 on T cells (Compare Supplementary Figure S4A with Figure 3B), and both CD103+ and CD103- CD8+ cells underwent proliferation in co-culture with HGSC (exemplified for PEA-1 in Supplementary Figure S4B).

Based on the known role of TGF-β in the induction of CD103 in mouse models and T cell clones [20, 21], we next assessed whether concurrent T cell activation and TGF-β receptor signaling was required for CD103 induction in PBMC:HGSC co-cultures (n≥3 donors). Induction of CD103 on PBMC was fully abrogated in the presence of TGF-β receptor I (TGFβR1) kinase inhibitor SB-431542 (Figure 3G) without affecting T cell proliferation (Figure 3H). To exclude an effect of selective depletion of CD8+ cells by TGFβR1 inhibition, we also analyzed the relative distribution of CD8 versus CD4 cells after treatment with either TGFβR1 kinase inhibitor alone, anti-CD3 agonistic antibody or the combination of both. While T cell activation with anti-CD3 agonist skewed the cell population towards a CD8+ phenotype, this change was not affected by treatment with TGFβR1 inhibitor (Supplementary Figure S4C). Finally, to confirm a causal role for TGF-β in the induction of CD103 on peripheral CD8+ T cells, we activated T cells in the presence or absence of recombinant TGF-β (rTGF-β1). Treatment with rTGF-β1 did not affect T cell proliferation as assessed by CFSE dilution, but induced a significant upregulation of CD103 (Supplementary Figure S4D and S4E). These data suggested that cancer antigen-specific circulating CD8+ T cells can upregulate CD103 after activation in the presence of HGSC cancer cells through a combination of TCR- and TGFβR1-signaling.

**Figure 4:**
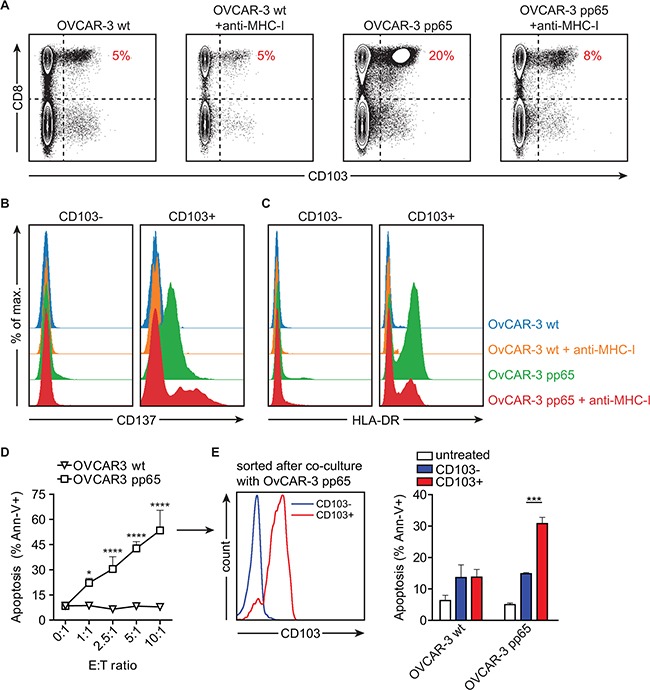
Activation in the presence of HGSC cell lines induces CD103 on CMV pp65-reactive CD8+ tumor-lytic T cells PBMCs from a CMV seropositive donor were isolated and cultured in the presence of OVCAR-3 wildtype (OVCAR-3 wt) cells or OVCAR-3 cells transduced with CMV pp65 (OVCAR-3 pp65) for 5 days (n=3). **A.** Representative flow cytometry images of CD103 expression on the CD8+ T cell and CD4+ T cell populations after co-incubation for 5 days with OVCAR-3 wt or OVCAR-3 pp65 alone or in the presence of MHC class I blocking antibody W6/32. **B.** Histograms representing CD137 or **C.** HLA-DR expression on CD103+ and CD103- CD8+ PBMCs after co-incubation for 5 days with OVCAR-3 wt or OVCAR-3 pp65 alone or in the presence of MHC class I blocking antibody W6/32. **D.** Percentage of apoptotic OVCAR-3 wt or OVCAR-3 pp65 tumor cells after co-culture with PBMCs for 5 days. **E.** Histograms representing CD103 expression on sorted CD103- and CD103+ cells after 5 day co-incubation with OVCAR-3 pp65 cells (left), and percentage of apoptotic OVCAR-3 wt or OVCAR-3 pp65 tumor cells after co-culture with sorted CD103- or CD103+ cells for 48 hours (right). Differences were assessed by Mann-Whitney U or one-way ANOVA tests.

To demonstrate that CD103 is indeed induced on cancer antigen-specific T cells during an ongoing antitumor immune response against HGSC, we employed a cytomegalovirus (CMV) model system. OVCAR-3 HGSC cells were transfected with the pp65 protein of CMV. Activation and antitumor activity were then assessed using PBMC from a healthy CMV-seropositive donor. After 5 days of co-incubation, CD103 was induced on ~20% of all CD8+ cells and these CD103+ cells (but not CD103- cells) co-expressed classical markers of T cell activation such as CD137 and HLA-DR (Figure 4A-4C). As observed earlier (Figure 3), PBMC co-cultured with wildtype OVCAR-3 cells in the absence of an activating signal did not induce CD103, nor did these cells upregulate CD137 and HLA-DR (Figure 4A-4C). Induction of CD103 and upregulation of CD137 and HLA-DR was abrogated by treatment with MHC class I blocking antibody W6/32, demonstrating antigen specificity (Figure 4A-4C). Of note, CD4+ T cells did not upregulate CD103 (Figure 4A), nor CD137, HLA-DR, and OX40 (not shown). Concomitant to the induction of CD103, T cells in these co-cultures induced specific apoptosis in pp65+, but not wildtype, OVCAR-3 cells (Figure 4D). Finally, isolation of CD103+ versus CD103- negative cells after 5 day co-culture revealed that the specific recognition and apoptotic capacity were restricted to the CD103+ cell population (Figure 4E). Taken together, these data indicate that CD103 is induced on polyclonal and polyfunctional cancer antigen-specific CD8+ T cells in response to specific recognition of HGSC cells in the presence of TGF-β produced by the epithelium.

### CD103+ TIL *in situ* are characterized by ongoing TCR- and TGFβR1-signaling

**Figure 5:**
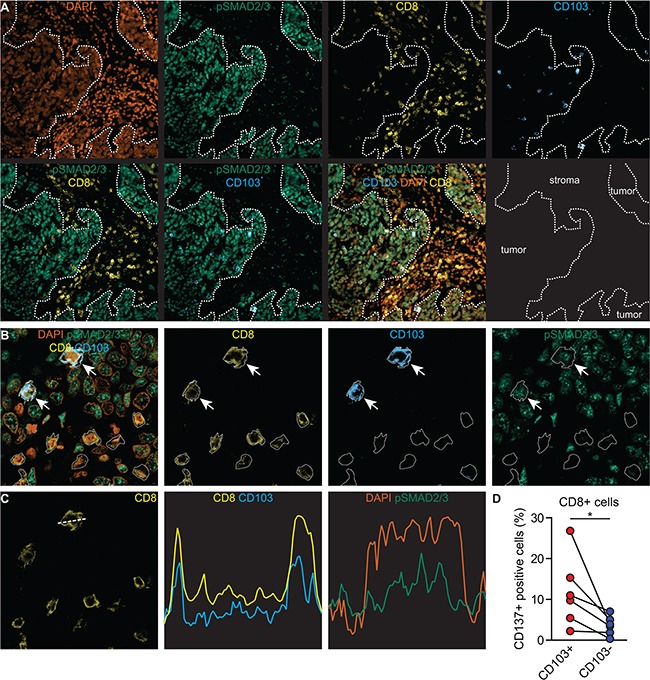
CD103+ TIL *in situ* are characterized by ongoing TCR and TGFβR1-signaling **A-C.** FFPEtissue slides from HGSC patients were used for immunofluorescent staining. **A.** Representative single and multichannel images of tissue from a patient with HGSC stained for DNA (orange), pSMAD2/3 (green), anti-CD8 (yellow) and anti-CD103 (blue). **B.** Representative single and multichannel images ofCD8+ CD103+ pSMAD2/3+ intraepithelial and CD8+ CD103- pSMAD2/3- stromal cells. CD8+CD103+ cells are depicted with an arrow. **C.** Representative image by confocal microscopy of a cross section of a CD8+ CD103+ T cell. CD8 and CD103 are expressed on the cell membrane whereas DAPI and pSmad2/3 are expressed within the nucleus. **D.** HGSC tumor tissue was subjected to enzymatic digestion and analyzed by flow cytometry. The percentage of CD137+ cells within the CD8+ CD103+ and CD8+ CD103- T cell populations is indicated. Differences were assessed by Mann-Whitney U tests.

To confirm this proposed mode of action for the ontogeny of CD103+ TIL *in situ*, we first assessed whether CD103+ TIL in HGSC tumor tissue demonstrated signs of active TGF-β signaling [22]. Hereto, paraffin-embedded tissue of HGSC was probed by fluorescent microscopy for simultaneous expression of CD8, CD103 and nuclear pSMAD2/3, a hallmark of TGF-β signaling. HGSC tumor epithelial islets were characterized by a pronounced nuclear expression of pSMAD2/3 (Figure 5A), a finding confirmed in a subsequent set of 37 HGSC patients by immunohistochemistry (Supplementary Figure S5A). Moreover, CD8+ CD103+ TIL, but not CD8+ CD103- TIL localized almost exclusively to these pSMAD2/3+ tumor islets (Figure 5A). Subsequent confocal analysis revealed that CD8+ CD103+ TIL were characterized by nuclear pSMAD2/3 expression, whereas CD8+ CD103- TIL were largely negative for pSMAD2/3 expression (Figure 5B, 5C and Supplementary Figure S5B-S5C). Of note, while a significant number of stromal cells in HGSC tumors expressed pSMAD2/3 in the nucleus, these cells were all negative for CD8 (Supplementary Figure S5C), suggesting a non CTL-origin.

To assess TCR signaling, we determined the expression of the marker for recent T cell activation CD137 [23] in several HGSC digests (n=6). In almost all cases, CD137 was expressed on a significantly higher fraction of CD103+ cells than on CD103- cells (Figure 5D, p=0.008). Taken together, our data suggest that CD103 expression on CD8+ TIL *in situ* is likely the result of concurrent TCR- and TGFβR1-signaling.

### CD103+ HGSC TIL dominantly express checkpoint molecules PD-1 and CD27

**Figure 6:**
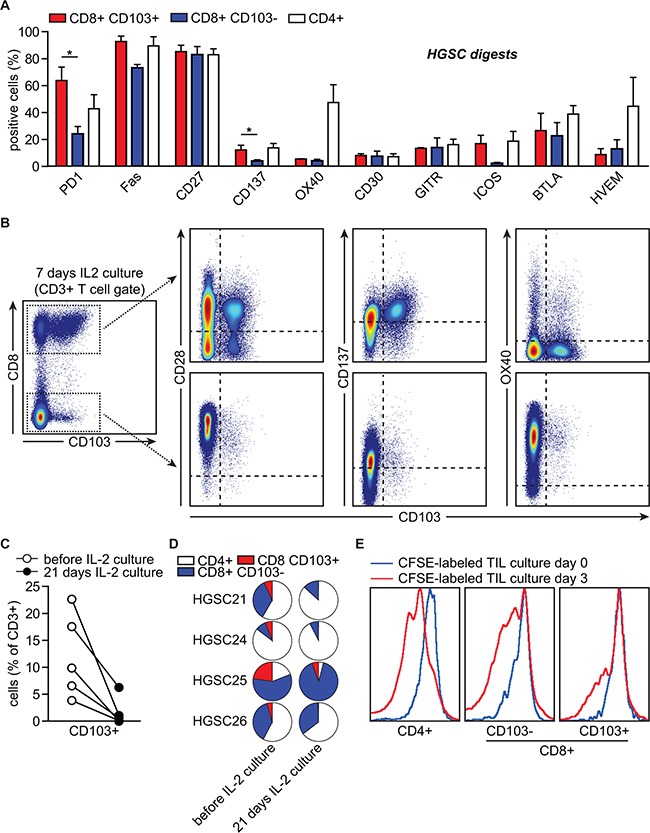
CD103+ TIL dominantly express checkpoint molecules PD-1 and CD27 HGSC tumor tissue was subjected to enzymatic digestion and analyzed by flow cytometry. **A.** Bar graph representing the percentage of cells positive for the indicated checkpoint molecules stratified by T cell population. **B.** Representative flow cytometry image of CD103 (x-axis) versus CD28, CD137 and OX40 (y-axis) (co-)expression within the CD8+ T cell (upper row) and the CD8- T cell population (lower row). **C.** Percentage of CD103 + cells within the CD3+ cell population after prolonged IL-2 culture (3 weeks). **D.** Pie charts representing percentage of indicated cell populations before and after prolonged IL-2 culture (3 weeks). TIL were derived from tumor digests (N=4). **E.** CFSE dilution showing proliferation of the indicated cell populations during IL-2 culture of TIL. Differences were assessed by Mann-Whitney U or one-way ANOVA.

Our data suggested CD8+ CD103+ T cells are an adaptive immune population infiltrating the epithelial bed of HGSC. Therefore, checkpoint molecules expressed on CD103+ cells may represent key targets for immunotherapy in HGSC. To assess this, we screened a series of HGSC digests (n≥3) for expression of clinically relevant antibody targets on CD8+ CD103+ and CD103- T cells. CD8+ CD103+TIL displayed a statistically significant higher percentage of PD-1+ cells when compared to their CD103- counterparts (Figure 6A, p=0.04). In addition, expression of the pro-apoptotic molecule Fas was higher in CD103+ cells (Figure 6A, p>0.05), in line with a recent report on a FasL-mediated endothelial barrier in epithelial islets in HGSC [24]. Immune checkpoint molecules TIM-3, LAG-3 and CTLA-4 could not be detected in HGSC digests (not shown). Expression of clinically relevant immune checkpoints from the tumor necrosis factor (TNF) family was largely restricted to expression of CD27, although no differences were observed between CD103+ and CD103- cells (Figure 6A). Finally, as described above, CD103+, and to a significantly lesser extent, CD103- cells expressed the marker of recent T cell activation CD137 (Figure 6A). To confirm the dominant co-expression of CD27 on CD103+ CD8+ TIL, we analyzed serial sections of our patient cohort (n=186; see also Figure 1). As anticipated based on our flow cytometric analysis of CD27 expression, both epithelial CD27+ and stromal CD27+ cells could be observed (Supplementary Figure S6A). Epithelial CD27+ cells co-localized with CD103+ cells and epithelial CD8+ cells in the adjacent sections (Supplementary Figure S6A). Analysis of the number of cells revealed a striking correlation between total CD103+ cells and epithelial CD8+ and epithelial CD27+ cells (Supplementary Figure S6B). Taken together, these data identify PD-1, CD137 and CD27 as key targets for therapeutic reactivation of intraepithelial T cells in HGSC.

### CD103+ HGSC TIL poorly expand during standard IL-2 culture for adoptive T cell therapy

We speculated that the prolonged contact of CD103+ CD8+ TIL with the cancer islets might render these cells less fit to expand on IL-2. Therefore, we assessed whether CD103+ TIL could be expanded from HGSC digests using a standard culture protocol of 6000 U/mL of IL-2 (n=6). After 7 days, CD103+ T cells were readily detected in IL-2 cultures and co-expressed CD8, but not CD4 (Figure 6B). Similar to the HGSC digests, CD8+ CD103+ cells expressed CD137 and were negative for OX40, whereas CD4+ TIL expressed high levels of OX40 at the cell surface (Figure 6B). Notably, prolonged IL-2 culture (3 weeks) resulted in a significant loss of CD103+ T cells from the TIL product (Figure 6C). Indeed, analysis of 4 distinct TIL cultures revealed a predominant expansion of CD4+ and/or CD8+ CD103- cells over CD8+ CD103+ cells (Figure 6D). In line with this, CD8+ CD103+ cells underwent less rounds of cellular proliferation during IL-2 culture compared to CD8+ CD103- and CD4+ cells (Figure 6E). Taken together, these data highlight a defective expansion of intraepithelial CD8+ TIL in a clinically relevant IL-2 expansion culture.

## DISCUSSION

In the current study we demonstrate that CD103+ T cells in HGSC are classical TCRαβ+ CD8αβ+ T cells. These cells are generated as a consequence of contact between tumor-infiltrating CD8+ T cells and the epithelial micro-environment. In addition, we confirm the previously published prognostic benefit of total CD103+ TIL in HGSC, but demonstrate that this benefit is restricted to patients receiving primary cytoreductive surgery (PS). Our data further suggest that CD103+ TIL could be effectively targeted by dual PD-1/CD27 checkpoint modulation. Finally, we propose that novel expansion protocols for TIL from HGSC are likely required to maintain the CD103+ population for adoptive transfer.

Our data support the notion that CD103 defines CD8+ cells that are localized in the epithelial compartment of epithelium-derived tumors. Indeed, previous work in HGSC, non-small cell lung cancer (NSCLC), urothelial cell carcinoma of the bladder and endometrial cancer all suggest CD103+ TIL infiltration is restricted to epithelial cancer islets [7, 16, 25–27]. However, our study also highlights key differences in the observed phenotypes for CD103+ TIL between malignancies. In NSCLC, CD103+ TIL were found to be of a homogeneous CD69+ CD62L− CD28− CD27+ CD45RA+ CD45RO+ CCR7− subtype [16]. By contrast, we recently found that CD103+ TIL in endometrial cancer were of a CD45RA-CD45RO+CCR7+/− phenotype [27]. Here, we demonstrate that in HGSC CD103+ TIL were homogeneously CD3+ CD56- TCRαβ+ CD8αβ+ CD4- CD45RO+, but heterogeneously expressed differentiation markers CCR7, CD27, and CD28. Furthermore, we demonstrate that almost all peripheral blood CD8+ T cells rapidly upregulate CD103 after activation in the presence of HGSC cancer cells in a TGF-β-dependent manner. These data suggest that CD103+ TIL are formed as a consequence of an adaptive immune response – comprising T cells at various stages of differentiation – instead of an expansion of T_RM_. In line with this, short-term culture of tumor digests with IL-2 revealed a heterogeneous expression of CD28 on CD103+ and CD103- TIL.

Based on our data and several recent publications on T cell infiltration and migration in(to) ovarian tumors [7, 11, 28, 29], we therefore propose a mode of action for the generation of CD103+ T cells. CD8+ T cells from the peripheral blood extravasate across the tumor vasculature and likely arrive in the small fibronectin/collagen-rich stromal areas that surround the cancer islets. This assumption is supported by the observation that the density of CD8+ TIL in stromal areas of most HGSC tumors is considerably higher than in the neighboring epithelial tissue, suggesting CD8+ T cell migration into cancer islets is a limiting factor (this paper and [28]). Herein, expression of e.g. FasL on the tumor vasculature is likely to play a role in limiting CD8+ T cell migration [24]. After extravasation, collagen density and fiber structure likely function as a barrier for effective CD8+ TIL migration from stromal areas into the epithelium of ovarian tumors [28]. In HGSC tumors where the collagen adopts a non-ring-like open structure, CD8+ TIL can cross the stromal barrier and engage epithelial HGSC cells [28]. At this point, cancer cell-reactive CD8+ T cells likely engage peptide-MHC class I molecules on the epithelial cancer cell and T cell receptor (TCR) signaling is triggered. Concurrently, epithelial cell-secreted TGF-β triggers TGF-β receptor signaling in the CD8+ T cell, resulting in upregulation of CD103. In line with this, we observed several CD103+ TIL in confocal analyses on the border of stromal and epithelial areas. These TIL showed signs of migration into the epithelium as evidenced by the characteristic elongation of activated T cells. If the T cell:cancer cell interaction results in release of e.g. IFN-y, known immunosuppressive factors like PD-L1 are likely to be (transiently) upregulated, effectively shutting down the T cell. Reversing this immunosuppression by means of checkpoint inhibition and/or T cell agonistic antibodies is likely to reactivate the infiltrating CD103+ TIL and mediate cancer cell eradication.

Our data highlight three interesting immune-modulating targets to achieve this, namely PD-1, CD137 and CD27. Predominant co-expression of PD-1 on the CD103+ TIL in HGSC has previously been reported and may be a consequence of TCR signaling occurring more frequently/exclusively in CD103+ TIL compared to CD103- TIL [29]. In line with this supposition, we further report that the marker for recent T cell activation CD137 was also expressed in a higher fraction of CD103+ TIL than in CD103- TIL. Interestingly, both PD-1 and CD137 have been reported to define the tumor-reactive T cell repertoire and our data show both markers are largely expressed within the CD103+, but not CD103- TIL populations [23, 30]. In depth analysis of the TCR repertoire of both subpopulations therefore appears warranted. Further, combinatorial immunotherapeutic strategies that target PD-1, CD137 and/or CD27 are likely to provide meaningful clinical benefit to HGSC patients with a high number of CD103+ TIL. Several trials in this respect are ongoing and it will be interesting to see if CD103 can herein be employed as a treatment-response biomarker.

Another interesting aspect of our analysis of CD103+ TIL concerns their minimal capacity for expansion under standard IL-2 culture conditions. Several trials on adoptive T cell transfer in ovarian cancer have been conducted with minimal observed clinical benefit [31, 32]. Our data provide a possible explanation by showing that the infusion product obtained after expansion is likely reduced in the intraepithelial (tumor-reactive) CD103+ TIL population and predominantly comprised of CD4+ and stromal CD8+ T cells. Of note, such a differential expansion of TIL subsets has previously been reported in the context of rapid TIL expansion for clinical infusion using artificial antigen presenting K562 cells (K562 aAPC) [33]. aAPC that did not produce cytokines, or were transduced to secrete IL-2, preferentially expanded CD4+ cells, most of which were FoxP3+ Treg cells. By contrast, aAPC that secreted IL-21 promoted a significantly more expansion of CD8+ cells, while minimizing Treg expansion. Analogously, it is conceivable that the exposure to TGF-β primes CD8+ CD103+ TIL for differential responses to cytokines when compared to CD8+ CD103- TIL. Novel expansion protocols that incorporate alternative cytokines to IL-2 may therefore provide a way of expanding CD103+ TIL for therapy.

A caveat of our study is the lack of functional data on the anti-tumor effects of CD103+ and CD103- T cell populations in an autologous patient-derived xenograft (PDX) model. While we established PDX from several of the patients included in this study [34], we were unable to expand the low numbers of CD3+CD56-TCRαβ+CD8αβ+CD4-CD103+ cells obtained after isolation by fluorescence-activated cell sorting. Indeed, culture of isolated TIL in the presence of IL-2 resulted in expansion of sorted CD3+ CD56- TCRαβ+ CD4+ and CD3+ CD56- TCRαβ+ CD8αβ+ CD4- CD103- cells, but not CD3+ CD56- TCRαβ+ CD8αβ+ CD4- CD103+ cells in 10/10 samples (data not shown). These data are in line with our results on the loss of CD103+ TIL from IL-2 cultures as depicted in Figure 5.

Finally, we observed clear differences in the prognostic benefit of stromal and epithelial CD8+ and CD103+ cells in relation to the treatment regime (Supplementary Figure S1). Stromal CD8+ T cells were of no prognostic benefit in any of the patient groups analyzed, nor were total CD8+ cells. By contrast, epithelial CD8+ cells and total CD103+ cells were of prognostic benefit only in patients treated by primary surgery and adjuvant chemotherapy. Patients treated in the neo-adjuvant setting did not benefit from CD8+ or CD103+ cell infiltration. Why patients treated in the neo-adjuvant setting do not benefit from T cell infiltration remains unclear, but may be related to underlying differences in the biology of these cancers [35] or to changes in the tumor microenvironment induced by chemotherapy [36].

In conclusion, our data indicate CD103+ TIL in HGSC are formed as the result of an adaptive anti-tumor immune response against HGSC. Combined checkpoint modulation that targets PD-1, CD137 and/or CD27 should be explored for treatment of HGSC patients with high numbers of CD103+ TIL.

## MATERIALS AND METHODS

### Patients and ethics

The patient cohort used for retrospective analysis of CD3, CD8 and CD103 infiltration has been reported previously [12]. In brief, patients with advanced stage (FIGO stage IIb) high-grade serous ovarian cancer were selected for analysis (N=186). 84 patients received primary surgery (PS) and 102 patients received neoadjuvant chemotherapy (NACT). All patient data was placed in an anonymous database, in which patient identity was protected by unique patient codes. According to Dutch law, no approval from our institutional review board was needed. Primary patient TIL used for prospective analysis of CD103+ TIL phenotype were isolated from fresh tumor samples obtained during cytoreductive surgery. Formalin-fixed paraffin-embedded (FFPE) tissue was available from several patients for matched analysis. This material was obtained from surgical tumor waste for which patients gave informed consent. No additional approval from our institutional review board was needed under Dutch law.

### Immunohistochemistry

Preparation of sections, antigen retrieval and blocking was performed as described previously [12]. Sections were incubated with either rabbit-anti human CD103 mAb (anti-αEβ7-integrin, Abcam, Cambridge, UK, 1:200 in blocking buffer), mouse-anti human CD8 mAb (DAKO, Heverlee, Belgium, clone C8/144B, 1:25 in blocking buffer), mouse-anti human CD3 mAb (DAKO 1:25 in blocking buffer) or rabbit anti-human pSMAD2/3 (D27F4, Cell signaling, 1:50 in blocking buffer) overnight at 4°C. Slides were subsequently incubated with a peroxidase-labeled polymer (Envision+ anti-rabbit or anti-mouse; DAKO). For CD103, a Biotin Tyramide working solution was used according to the manufacturer's instructions (TSA Kit Perkin Elmer, Waltham, USA 1:50) and slides were incubated with streptavidin-HRP (TSA kit, Perkin Elmer, Waltham, USA 1:100). Specific signal for all staining was visualized by 3,3'diaminobenzidin (DAB). Slides were counterstained with hematoxylin. For each core the percentage of tumor/stromal surface was estimated based on consecutive H&E slides using semi-quantitative estimation. The total number of cells that stained positive for CD103 was counted per core and corrected for the total surface area of the core (epithelium+stroma). For CD3+ and CD8+ cells, the intra-epithelial and stromal cells were counted separately and corrected for the surface area of each individual area. For epithelial counts patients were included if at least two cores contained >20% tumor epithelium. For stromal counts, patients were included if at least two of the cores contained >10% stroma and >20% tumor epithelium. For pSMAD2/3 staining, epithelial and stromal regions of individual cores were scored for no (0), low (1), intermediate (2) or high (3) expression and the sum of all 3 cores per patient used as the final score. For whole tumor sections, three individual 1 mm^2^ regions of tumor epithelium were selected and counted. All slides were counted manually by two individuals that were blinded for clinicopathological data. The two individual scores were compared, and differences in counts of over 10% were reanalyzed until consensus was reached. Epithelial CD27+ cell counts were reported previously [12].

### Immunofluorescence

Preparation of sections, antigen retrieval and blocking was performed as described previously [12]. Sections were incubated overnight with rabbit-anti human CD103 mAb, washed and incubated with Envision-HRP anti-rabbit followed by fluorophore tyramide amplification diluents (TSA KIT Perkin Elmer, MA, USA 1:50) according to the manufacturer's instructions. Next, slides were incubated overnight at 4°C with mouse anti-human CD8 (DAKO, Heverlee, Belgium, clone C8/144B, 1:25 in blocking buffer) and rabbit anti-human pSMAD2/3 (D27F4, Cell signaling, 1:50 in blocking buffer). Sections were subsequently incubated with goat-anti-mouse Alexa Fluor 555 (Life Technologies, Eugene, OR, USA GαM-AF555, 1:150) and goat-anti-rabbit Alexa Fluor 488 (Life Technologies, Eugene, OR, USA 1:150). Nuclei were visualized with DAPI. Sections were embedded in prolong Gold anti-fade mounting medium (Life Technologies, Eugene, OR, USA) and scanned using a TissueFaxs imaging system (TissueGnostics, Vienna, Austria). Processed channels were merged using Adobe Photoshop. Analysis was performed using Fiji [37].

### Tumor digests and TIL cultures

Fresh tumor material was obtained from patients undergoing cytoreductive surgery (n=20). With a scalpel, tumor pieces of approximately 1 mm^3^ were cut, and subjected to enzymatic digestion (RPMI supplemented with 1 mg/ml collagenase type IV (Life technologies) and 31 U/ml rhDNase (Pulmozyme, Genentech, California, USA)) for 30 minutes at 37°C or overnight at room temperature. Subsequently, the digestion medium containing remaining tumor pieces was filtered over a 70 μm cell strainer (Corning, Amsterdam, The Netherlands). For phenotypic analyses, cells were pelleted, washed, and cryopreserved until further use. For TIL culture, cells were pelleted, washed and cultured in AIM-V medium containing 5% pooled human AB serum (Life Technologies) and 6000 IU/ml IL-2 (PROLEUKIN, Novartis). TIL cultures were routinely phenotyped using the antibodies described below. For proliferation assays, TIL cultures were labeled using the CellTrace™ CFSE Cell Proliferation Kit for flow cytometry (Life Technologies) according to the manufacturer's instructions.

### Flow cytometric analysis

CD103+ TIL phenotype was characterized by multiparameter flow cytometry in combination with fluorescence-activated cell sorting (FACS). The Zombie Aqua Fixable Viability Kit (BioLegend, Uithoorn, The Netherlands) was used for live/dead staining according to the manufacturer's instructions. Antibodies used for analysis and cell sorting of CD103+ cells in HGSC tumor digests were CD3-BV421 (OKT3), CD103-FITC (ber-ACT8), CD56-PE (clone CMSSB) (all BD Biosciences, Ettenleur, The Netherlands), CD4-PerCP-CY5.5 (OKT4), CD8β-PE-Cy7 (clone SIDI8BEE), TCRαβ-APC (IP26) and CD8α-APC-eFluor780 (RPA-T8) (all eBioscience, Vienna, Austria). Flow cytometry and cell sorting was performed on a Beckman Coulter MoFlo Astrios (BD Biosciences). For analysis of T cell differentiation in HGSC digests a combination of CCR7-BV421 (clone 150503), CD103-FITC (ber-ACT8) (both BD Biosciences), CD27-APC (M-T271), CD45RO-PE-Cy7 (UCHL1), CD28-PerCP-Cy5.5 (CD28.2), CD3-PE (OKT3), CD8α-APC-eFluor780 (RPA-T8) (all eBioscience) and CD27-FITC (PeliCluster F) (Sanquin PeliCluster, Amsterdam, The Netherlands) were used. For analysis of expression of checkpoint molecules and costimulatory TNF family members in HGSC digests the following antibodies were used: CD134(OX40)-PE-Cy7 (ACT35), CD270(HVEM)-Alexa Fluor 647 (clone 94801), CD278(ICOS)-BV421 (DX29), CD272(BTLA)-APC (J168-540), CD30-PE (clone 550041) (all from BD Biosciences), CD357(GITR)-PE-Cy7 (eBioAITR), CD137-PE (4B4), PD1-APC (MIH4), CD95-PE-Cy7 (DX2), CD28-PerCP-Cy5.5 (CD28.2) (all from eBioscience) and CD27-FITC (PeliCluster F) (Sanquin PeliCluster). For analysis of T cell activation, HLA-DR-BV421 (G46-6) CD103-FITC (ber-ACT8) (both BD Biosciences), CD137-PE (4B4), CD3-PerCP-CY5.5 (OKT3), CD134(OX40)-PE-Cy7 (ACT35) and CD8α-APC-eFluor780 (RPA-T8) were used (all eBioscience). Flow cytometry was performed on a BD FACSVerse and samples were analyzed with Cytobank software (cytobank.org). For phenotyping of TIL cultures, the aforementioned antibodies were used in various combinations as indicated in the manuscript, acquired on a BD Accuri C6 flow cytometer (BD Biosciences) and analyzed using the accompanying software.

### Coculture assays for induction of CD103

Freshly isolated PBMCs were activated with purified anti-CD3 monoclonal antibody OKT3 (1:1000, eBioscience) in the presence or absence of HGSC-cell lines PEA-1, PEA-2, PEO-14, PEO-23, OVCAR-3 (all routinely profiled using STR and mycoplasm screening assays). Cell lines were cultured using RPMI supplemented with 10% FCS, 1% Glutamine and 1% Sodium pyruvate for PEA-1, PEA-2, PEO-14 and PEO-23, and RPMI supplemented with 10% FCS for OVCAR-3. All co-culture experiments were performed in AIM-V medium supplemented with 5% pooled human AB serum. Where indicated, cells were additionally incubated with 40 μM TGFβR1 inhibitor SB-431542 (Sigma-Aldrich, Zwijndrecht, The Netherlands) or recombinant TGFβ (Peprotech, Rocky Hill, United States). For proliferation assays, PBMC were labeled using the CellTrace™ CFSE Cell Proliferation Kit for flow cytometry (Life Technologies) according to the manufacturer's instructions. For analysis of antigen-specific immunity, OVCAR-3 cells were transfected with CMV pp65 protein using the pCMV6-pp65 Origene expression vector (VC101404-OR; Origene). Cells were cultured under G418 selection and used when >90% cells were pp65+ as assessed by antibody-based staining (IQ products clones C10/C11). PBMCs isolated from a CMV-seropositive donor (assessed by IgG antibodies to CMV) were co-cultured with pp65+ OVCAR-3 or wildtype OVCAR-3 cells for the indicated periods and effector-to-target (E:T) ratios and apoptosis assessed using Annexin-V staining (Immunotools). CD103+ cells induced after 5 days of co-culture were sorted using CD103-FITC (ber-ACT8) antibody and magnetic anti-mouse beads (Miltenyi Biotech) according to the manufacturer's instructions. Where indicated, cells were phenotyped using the antibody panel described above.

### Statistics

All statistical analyses were performed using IBM SPSS version 22 (SPSS Inc., Chicago, USA) or Graphpad Prism. Survival analysis was performed using the Kaplan-Meier method with Log Rank test to test for differences between groups. Groups were stratified on the basis of the highest tertile. Differences in cell infiltration between 43 matched ovarian and omental primary tumor tissues were assessed by Wilcoxon signed ranks test, no differences were found and therefore primary ovarian and omental tissues were used in the analyses. To determine differences in cell populations between clinicopathological variables or between different TIL subsets, the Mann-Whitney U or one-way ANOVA test were used. Spearman's rank correlation coefficient was used to determine correlation between epithelial and stromal CD3+ and CD8+ TIL and total CD103+ TIL. All tests were performed two-sided, and p-values <0.05 were considered significant.

## SUPPLEMENTARY FIGURES


