# Supplementary material for: CD103+ intraepithelial T cells in high-grade serous ovarian cancer are phenotypically diverse TCRαβ+ CD8αβ+ T cells that can be targeted for cancer immunotherapy
Source: Oncotarget. 2016 Sep 16;7(46):75130–44. doi: 10.18632/oncotarget.12077 (PMC5342728; doi:10.18632/oncotarget.12077)
Supplement: Supplementary file 1 [file oncotarget-07-75130-s001.pdf]

CD103+ intraepithelial T cells in high-grade serous ovarian cancer are phenotypically diverse TCRαβ+ CD8αβ+ T cells that can be targeted for cancer immunotherapy

SUPPLEMENTARY FIGURES

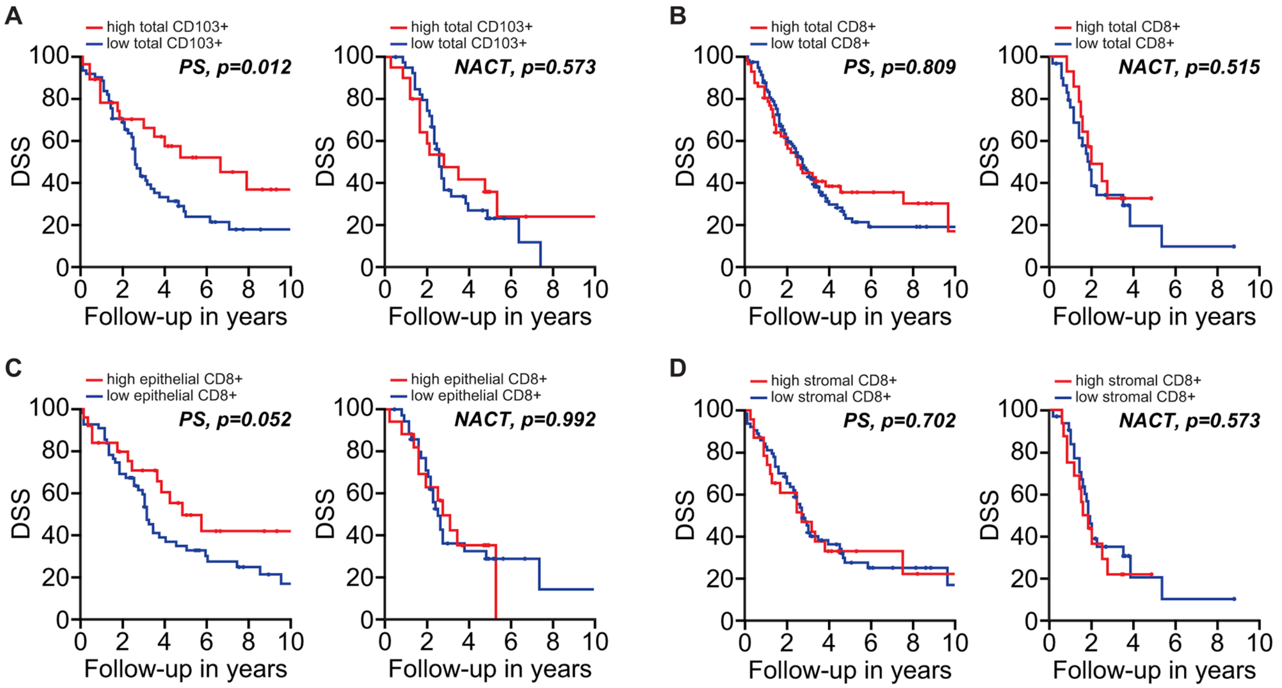

Supplementary Figure S1: CD103+ TIL are associated with improved prognosis in patients with high-grade serous epithelial ovarian cancer (HGSC). A. Disease-specific survival (DSS) (determined by Kaplan-Meier method with Log Rank test) of patients within the primary cytoreductive surgery (PS) and neo-adjuvant chemotherapy (NACT) cohort according to infiltration of total CD103+ cells. **B.** DSS of patients within the PS and NACT cohort according to infiltration of total CD8+ cells. **C.** DSS of patients within the PS and NACT cohort according to infiltration of epithelial CD8+ cells. **D.** DSS of patients within the PS and NACT cohort according to infiltration of stromal CD8+ cells.

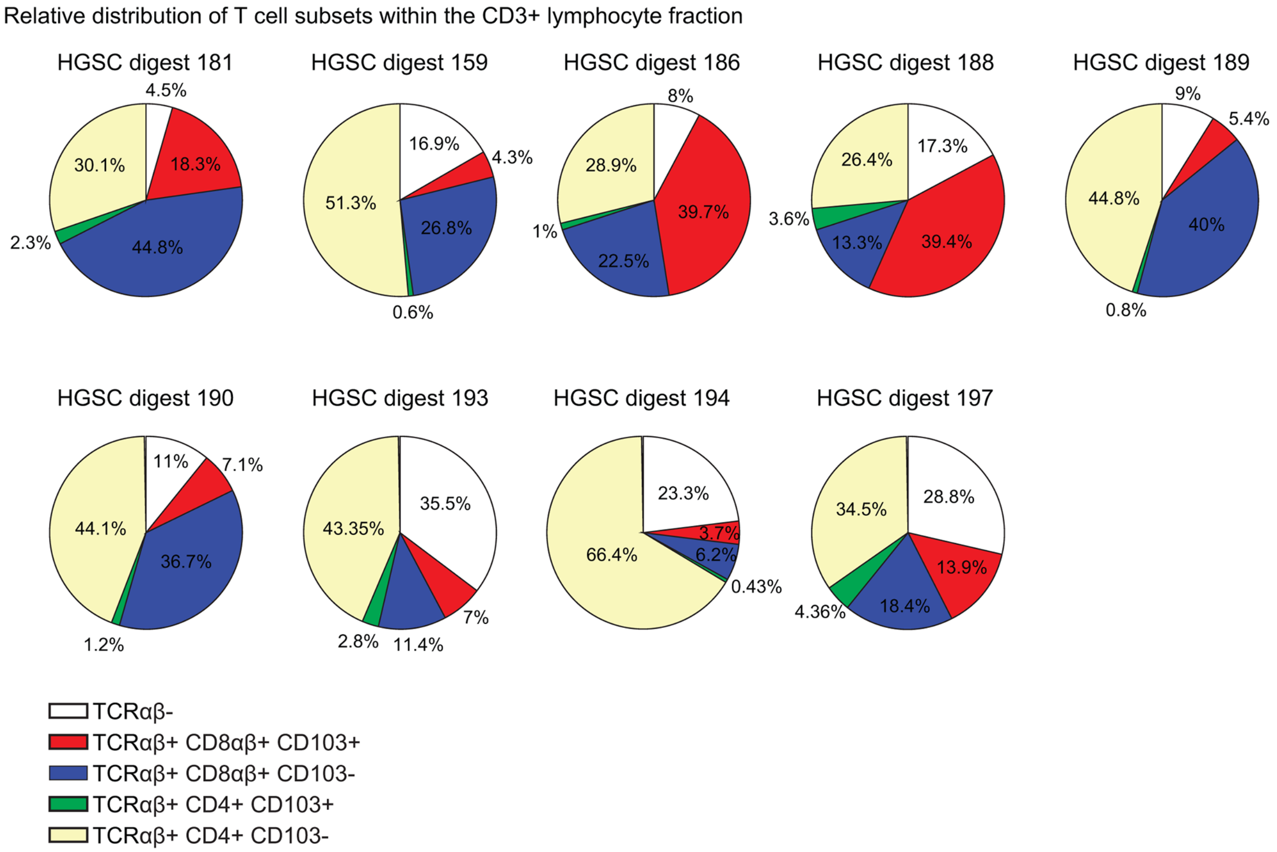

Supplementary Figure S2: Relative distribution of T cell subsets of each individual patient from Figure 2. HGSC tumor tissue was subjected to enzymatic digestion and analyzed by flow cytometry. Digests were stained using Zombie Aqua live/dead stain and antibodies against CD3, CD56, TCRαβ, CD8α, CD8β, CD4 and CD103. Distribution of T cell subsets within the CD3+ lymphocyte fraction was determined and plotted as a pie chart for each individual patient.

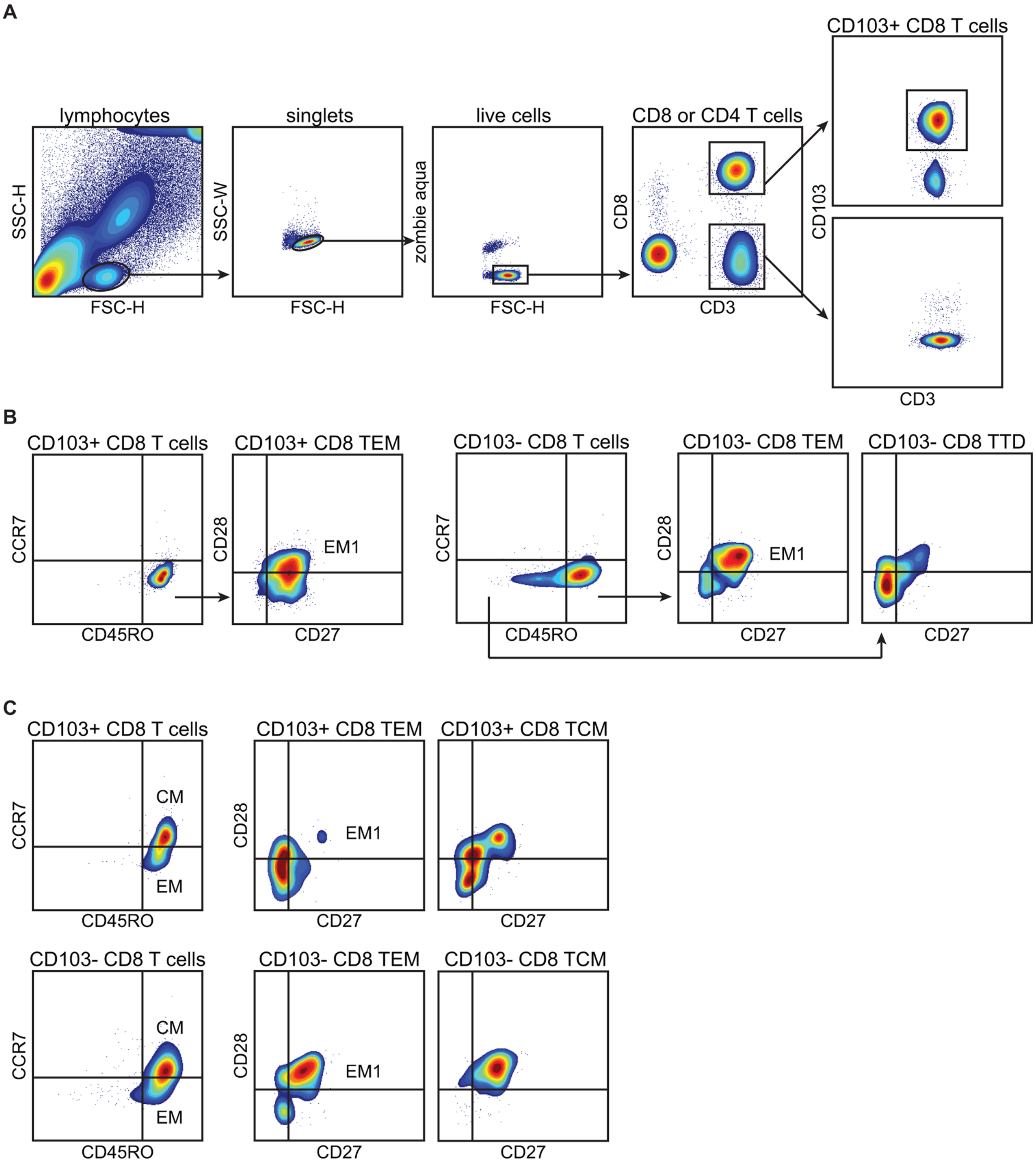

Supplementary Figure S3: Flow cytometric plots and gating strategy. HGSC tumor tissue was subjected to enzymatic digestion and analyzed by flow cytometry. **A.** Gating strategy for identifying live CD3+ CD8+ CD103+ and CD3+ CD8+ CD103- T cells. **B.** Flow cytometric plots for HGSC tumor digest #202 used in the quantification depicted in Figure 2G of the manuscript. **C.** Flow cytometric plots for HGSC tumor digest #207 used in the quantification depicted in Figure 2G of the manuscript. T_{CM}: central memory T cell. T_{EM}: effector memory T cell. T_{TD}: terminally differentiated T cell.

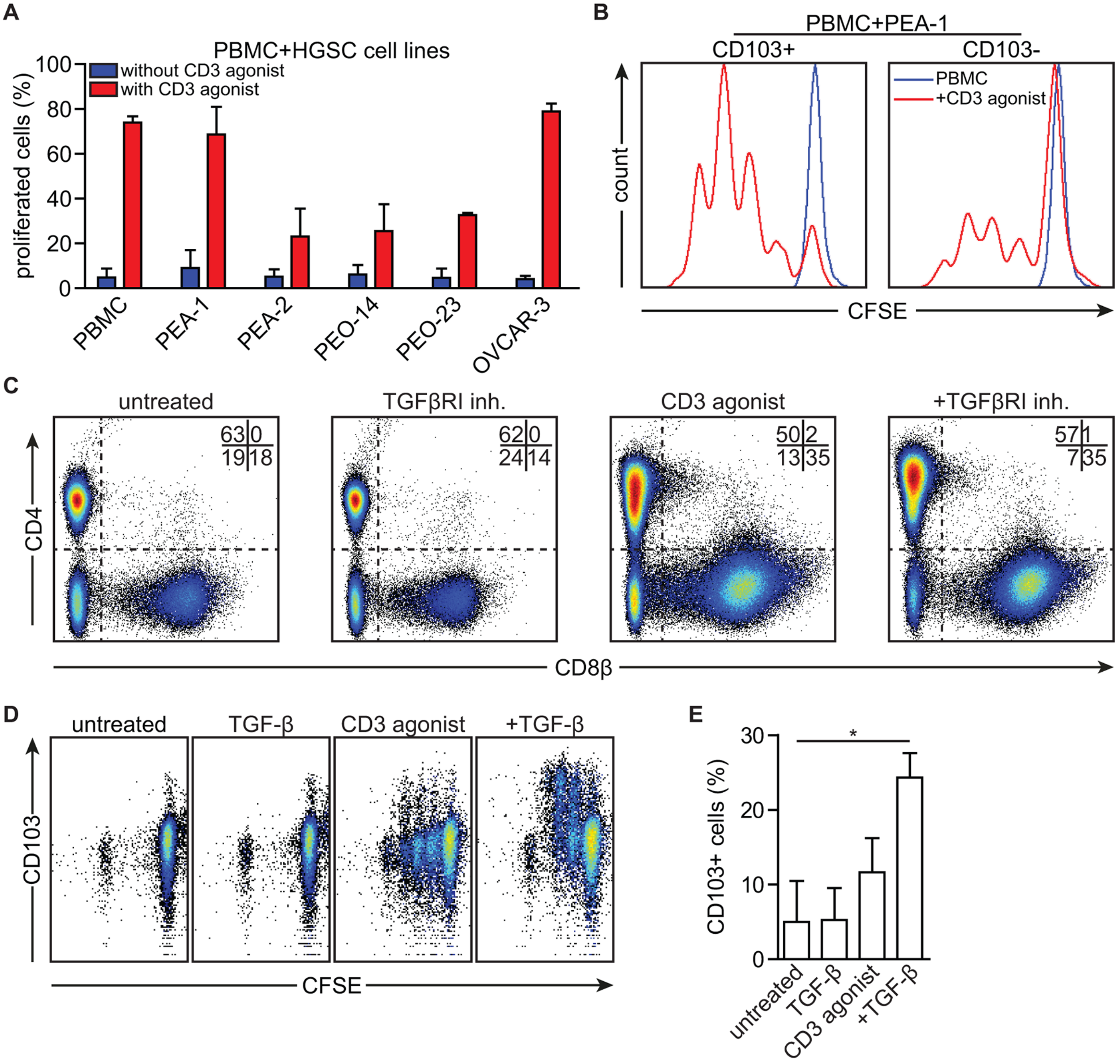

Supplementary Figure S4: Treatment with rTGF-β1 does not affect T cell proliferation but induces a significant upregulation of CD103 on T cells. Peripheral blood mononuclear cells (PBMCs) were isolated and cultured in the presence of HGSC cell lines. **A.** Bar graph representing the percentage of proliferating cells after incubation of PBMCs with HGSC cell lines (PEA-1, PEA-2, PEO-14, PEO-23, OVCAR-3) in the presence or absence of CD3 agonist. **B.** Representative histograms of T cell proliferation (CFSE dilution) after incubation of PBMCs with PEA-1 in the presence or absence of CD3 agonist within the CD103+ and the CD103- T cell population. **C.** Representative flow cytometry image of T cell phenotype after incubation of PBMCs with OVCAR-3 and TGFβR1 inhibitor and/or CD3 agonist as indicated. **D.** Representative images of CFSE dilution showing T cell proliferation after incubation of PBMCs with rTGF-β1 and/or CD3 agonist. **E.** Bar graph representing the percentage of CD103+ cells after incubation of PBMCs with rTGF-β1 and/or CD3 agonist. Differences were assessed by Mann-Whitney U or one-way ANOVA tests.

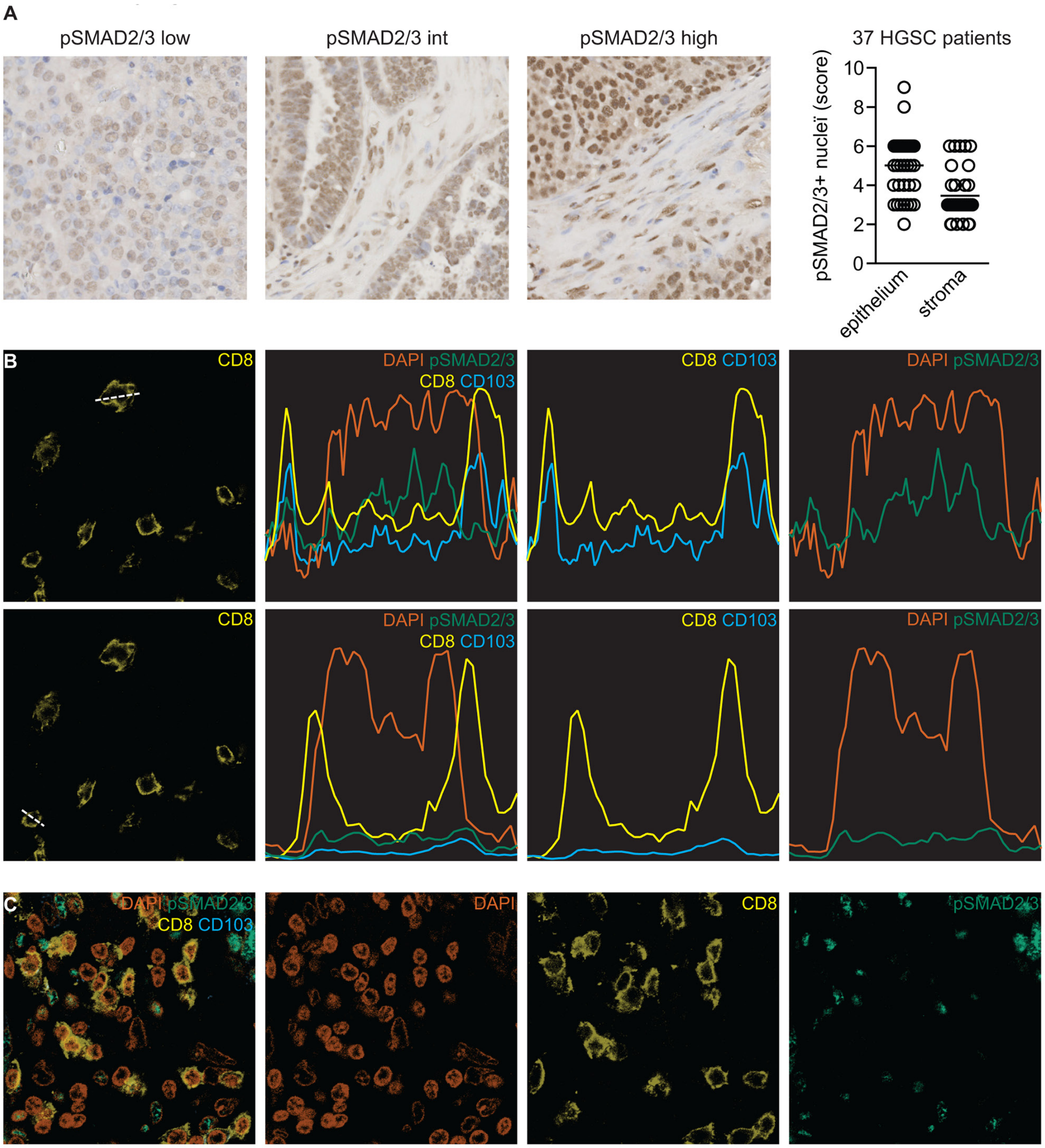

Supplementary Figure S5: CD103- CD8+ TIL are largely negative for pSMAD2/3 expression. A. HGSC tissue cores of 37 patients (3 cores per patient) were stained with anti-pSMAD2/3 antibody and scored for expression. **B/C.** Formalin-fixed paraffin-embedded (FFPE)tissue slides from HGSC patients were used for immunofluorescent staining. **B.** Representative images of cross sections of CD8+ T cells by confocal microscopy. Upper row shows an intraepithelial CD8+ CD103+ T cell with nuclear pSMAD2/3 expression. The lower row shows a stromal CD8+CD103- T cell where no pSMAD2/3 expression is observed in the nucleus. **C.** Representative single and multichannel images of tissue from a patient with HGSC stained for DNA (orange), pSMAD2/3 (green), anti-CD8 (yellow) and anti-CD103 (blue) antibodies.

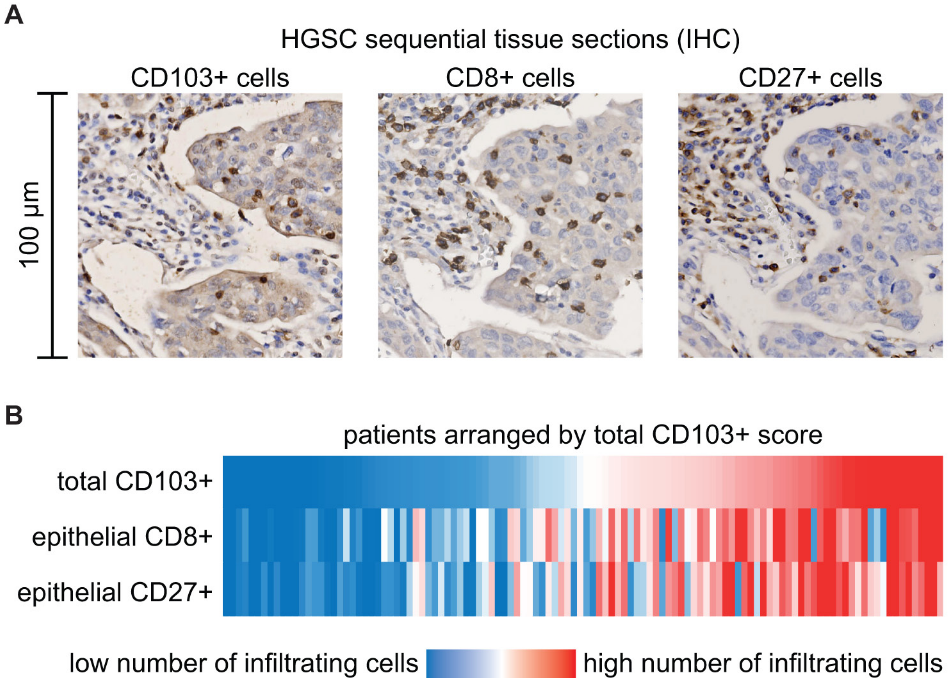

Supplementary Figure S6: CD103+ CD8+ TIL dominantly co-express checkpoint molecule CD27 **A.** Representative images of HGSC tissue cores with infiltration of CD103+, CD8+ or CD27+ cells. **B.** total CD103+, and epithelial CD8+ and CD27+ cells were quantified per patient (both cohorts). Patients were arranged from low to high total CD103+ cell infiltration and corresponding epithelial counts of CD8+ and CD27+ are displayed in a heatmap.
